# Identification of GPD1L as a Potential Prognosis Biomarker and Associated with Immune Infiltrates in Lung Adenocarcinoma

**DOI:** 10.1155/2023/9162249

**Published:** 2023-03-30

**Authors:** Zhengyang Fan, Song Wu, Hongyang Sang, Qianping Li, Shaofei Cheng, Hongling Zhu

**Affiliations:** ^1^Department of Cardiothoracic Surgery, Shanghai Sixth People's Hospital Affiliated to Shanghai Jiao Tong University School of Medicine, Shanghai, China; ^2^Department of Oncology, Shanghai Sixth People's Hospital Affiliated to Shanghai Jiao Tong University School of Medicine, Shanghai, China

## Abstract

Lung adenocarcinoma (LUAD) is one of the most prevalent pathological kinds of lung cancer, which is a common form of cancer that has a high death rate. Over the past several years, growing studies have indicated that GPD1L was involved in the advancement of a number of different cancers. However, its clinical significance in LUAD has not been investigated. In this study, following an examination of the TGCA datasets, we found that GPD1L displayed a dysregulated state in a wide variety of cancers; this led us to believe that GPD1L is an essential regulator in the progression of malignancies. In addition, we found that the expression of GPD1L was much lower in LUAD tissues when compared with nontumor specimens. According to the findings of ROC tests, GPD1L was able to effectively identify LUAD specimens from nontumor samples with an AUC value of 0.828 (95% confidence interval: 0.793 to 0.863). On the basis of the clinical study, a low expression of GPD1L was clearly related with both the N stage and the clinical stage. Moreover, based on the findings of a Kaplan-Meier survival study, elevated GPD1L expression was a strong indicator of considerably improved overall survival (OS) and disease-specific survival (DSS). GPD1L expression and clinical stages were found to be independent prognostic indicators for overall survival and disease-free survival in LUAD patients, according to multivariate analyses. Based on multivariate analysis, the C-indexes and calibration plots of the nomogram demonstrated an effective prediction performance for LUAD patients. Besides, the expression of GPD1L was positively related to mast cells, eosinophils, Tcm, TFH, iDC, DC, and macrophages, while negatively associated with Th2 cells, NK CD56dim cells, Tgd, Treg, and neutrophils. Finally, qRT-PCR was able to demonstrate that GPD1L had a significant amount of expression in LUAD. Additionally, according to the results of functional tests, overexpression of GPD1L had a significant inhibiting effect on the proliferation of LUAD cells. In general, the results of our study suggested that GPD1L had the potential to serve as a diagnostic and prognostic marker for LUAD.

## 1. Introduction

Over twenty-seven percent of all cancer-related deaths worldwide are attributable to lung cancer, with non-small cell lung cancer (NSCLC) being responsible for eighty percent of all lung cancer cases [1]. The histological subtype of non-small cell lung cancer that occurs most frequently is known as lung adenocarcinoma (LUAD) [2]. It has virtually reached the position of being the primary contributor to death among those living in urban regions of China [3]. Even with all of the advancements that have been made over the years in cancer diagnosis and treatment, the death rate of lung cancer is still rather high, which is particularly relevant to smokers [4, 5]. As a direct consequence of this, numerous patients who were diagnosed with early lung cancer did not receive adjuvant therapy following their surgeries [6, 7]. Consequently, the disease returned or spread to other parts of the body in some of the patients due to a number of variables, including that some people are diagnosed at an advanced stage of lung cancer [8, 9]. That may be one of the many reasons why this is the case. Besides, lung cancer patients do not receive an accurate picture of their prognosis through the use of the guided staging technique that is currently in place, which is another possible explanation [10, 11]. At this time, the histopathologic diagnosis and the neoplasm staging system are the only things that can accurately predict a patient's prognosis [12]. However, conventional approaches do not provide a precise enough picture of a patient's outlook to be used. In order to further aid doctors to treat LUAD, therefore, a trustworthy and precise marker for prognosis prediction needs to be established.

The protein known as glycerol 3-phosphate dehydrogenase 1-like (GPD1L) is encoded by the gene GPD1L, which is located on chromosome 3p22.3 [13]. This protein is responsible for catalyzing the conversion of sn-glycerol 3-phosphate to glycerone phosphate [14]. The GPD1L protein was discovered in the cytoplasm, and it was connected to the plasma membrane [15]. It was found in 2002 when the Mammalian Gene Collection (MGC) program of the National Institutes of Health attempted to find and sequence a cDNA clone [16]. Studies done in the past have found evidences that GPD1L was involved in more than one type of tumor. For example, Liu et al. discovered that the mRNA expression of GPD1L, which was found to be downregulated, and HIF1, which was found to be upregulated, exhibited a negative association (*r* = −.496, *p* = .001) in cT1-2 N0 head and neck squamous cell carcinoma (HNSCC) [17]. In addition to this, GPD1L has been shown to have a negative association with HIF1 expression and to be a factor that predicts lymph node metastases in cases of oral and HPV-related oropharyngeal cancer [18]. Zhao et al. showed that the expression level of GPD1L was low in colorectal cancer, and it had a strong correlation with the clinical stage, grade, and TNM stage of colorectal cancer [19]. In addition, GPD1L protein levels were also measured in HNSCC patients and found to be associated with a dismal prognosis for those patients with HNSCC [18]. However, on the other hand, very little is known about the function of GPD1L in LUAD.

Tumor microenvironment (TME) refers to the collection of cancer cells, immune cells, stromal cells, and extracellular matrix that together play a significant role in the progression of cancer [20]. Cancer cells are present in the TME, and these cells have the ability to infect neighboring tissues either directly or indirectly by traveling through blood and lymphatic channels [21]. These infiltrating cells have the capability of provoking an immune response through the release of cytokines and other substances that influence the growth of the tumor [22]. Growing researches have indicated that TME affected the procedures of tumor progression and have shown a possible predictive value for the clinical outcome of patients, including LUAD [23, 24].

The rapid growth of precision medicine has led to an increase in the number of studies in which researchers use statistical algorithms to investigate new diagnostic and therapeutic targets. The Cancer Genome Atlas (TCGA) delivered genomic profiles as well as clinical data, which made it feasible to study the association between genomic features and clinical as well as prognostic aspects. The purpose of this research was to investigate the clinical relevance of GPD1L in LUAD patients and to determine whether or not it was associated with immune cell infiltration.

## 2. Materials and Methods

### 2.1. Cell Lines and Cell Transfection

LUAD cell lines, including A549, H1299, HCC827, H226, and H23 cells, as well as normal bronchial epithelial cells (BEAS-2B), were purchased from the American Type Culture Collection (ATCC, Manassas, VA, USA). Next, the LUAD cell lines were cultured in a 5% CO_2_ incubator at 37°C in RPMI-1640 medium (Cat#11875119, Gibco, Shanghai, China) containing 10% fetal bovine serum (FBS) (Cat#12664025, Gibco, Shanghai, China). After mixing for 20 minutes at room temperature, 2 *μ*g of the overexpress plasmids targeting GPD1L in 100 *μ*l of RMPI 1640 media were combined with *μ*l of lipofectamine 3000 (Cat#L3000001, Invitrogen, Shanghai, China) that had been diluted in 100 *μ*l of RMPI 1640 media. Following a transfection time of 48 hours, the cells were harvested to carry out the following experiments.

### 2.2. Quantitative Real-Time PCR (qRT-PCR)

Trizol reagents (Cat#15596026, Invitrogen, MA, USA) were employed to isolate the total RNAs from various tissues and cells, and the concentration of total RNAs was examined by NanoDrop 2000 device (Cat#ND-2000-GL, Thermo, Waltham, MA, USA). Subsequently, 2 *μ*g of total RNAs was subjected to reverse reaction using EpiNext Hi-Fi cDNA Synthesis kit (AmyJet, Wuhan, Hubei, China) to obtain the cDNAs. Then, the qRT-PCR assays were carried out with the use of PrimeScriptTM RT Master Mix kits, which were purchased from Takara company (Dalian, Liaoning, China), and the Bio-Rad CFX96 PCR System (Bio-Rad, CA, USA). The relative expression levels were determined using the 2^−△△Ct^ method, with GAPDH serving as the control for the standardization process. The primer sequence for GPD1L was listed as follows: F-primer: 5′ATCAAGGGCATAGACGAGGG3′; R-primer: 5′TCTGCATCATCAACCACGGTA3′. The primer sequence for GAPDH was: F-primer: 5′TCAAGCTCATTTCCTGGTATGAC3′; R-primer: 5′CTTGCTCAGTGTCCTTGCTG3′.

### 2.3. Cell Viability Assay

The Cell Counting Kit-8 (CCK-8) test kits (Cat#HY-K0301, MedChemExpress, Shanghai, China) was utilized in order to determine the cellular proliferation of H1299 and H226 cells with overexpressing GPD1L. The cells were firstly inserted onto 96-well plates (2500 cells per well, 100 *μ*l) after GPD1L-overexpressing plasmids were transfected. Afterwards, the cells were applied for CCK-8 assays at 24, 48, 72, and 96 hours. After adding 10 *μ*l of CCK-8 reagents from MedChemExpress company (Cat#HY-K0301, Shanghai, China) and allowing the mixture to incubate for one hour, the optical density was measured using a microplate reader from BioTek (BioTek, Winooski, VT, USA) at a wavelength of 450 nm.

### 2.4. Data Collection and Processing

The TCGA Data Portal (http://www.tcgaportal.org/) was mined for the high-throughput gene expression data in order to collect it. This data was obtained not just from LUAD tissues but also from normal lung tissues in the TCGA Data Portal. Besides, these RNA-seq data (HTSeq-count) were obtained through the data portal of the Genomic Data Commons (GDC), which is open to the general public (https://gdc.cancer.gov/). The Illumina HiSeq RNA-seq platform was the source of these data, which included 535 LUAD samples in addition to 59 noncancerous samples.

### 2.5. Identification of the Aberrantly Expressed Genes in LUAD

In order to determine which genes were differentially expressed, the expression patterns of LUAD tissues and normal tissues were compared using R software. The edgeR Bioconductor package was used to undertake an investigation on the differential expression of particular genes. For the purpose of differentially expressed genes (DEGs) identification, the threshold values were determined to be |log2(fold change [FC])| greater than 2, *p* value less than 0.01, and false discovery rate (FDR) less than 0.01.

### 2.6. GPD1L Differential Expression in Pan-Cancer in the TCGA Database

In order to calculate the differential expression of GPD1L, boxplots and scatter plots were produced using the disease state as the variable. The illness condition was either tumor or normal. Receiver operating characteristic (ROC) curves were utilized to create an estimate of GPD1L's diagnostic performance. The statistical ranking for GPD1L expression that was designated as GPD1L high or GPD1L low, respectively, was determined by whether it was above or below the median value.

### 2.7. Prognostic Analysis

In order to determine the overall survival (OS) of patients who were part of the TCGA cohort, a Kaplan-Meier analysis was carried out. Univariate Cox regression analyses were carried out to determine the importance of GPD1L in evaluating overall survival (OS) and disease-specific survival (DSS) in patients with LUAD.

### 2.8. Analysis of DEGs between GPD1L High and Low Expression LUAD Groups

The unpaired Student *t*-test that is included in the DESeq2 (3.8) package was used to find differentially expressed genes (DEGs) comparing patients with high and low levels of GPD1L in the TCGA datasets.

### 2.9. Functional Enrichment Analysis

Disease Ontology (DO) enrichment analyses were carried out on DEGs with the help of the “clusterProfiler” and DOSE packages in the R programming language [25, 26]. The “clusterProfiler” R package was used to carry out the analyses based on DEGs that were conducted by GO and KEGG.

### 2.10. Nomogram Construction

Combining the findings of the genetic risk score model with clinical characteristics led to the development of a nomogram that was able to accurately forecast 3- and 5-year LUAD overall survival (OS). Calibration plots were used to evaluate the nomogram's ability to make accurate predictions. The area under the curve (AUC) was employed to analyze the time-dependent sensitivities and specificities of the nomogram for both the 3-year and 5-year OS ROC curves. R software was used as the statistical program for all of the studies that were done (version 3.4.1). The rms package of R software was used to construct the nomogram and calibration plots, and the timeROC package was used to conduct the analysis of the time-dependent ROC curve. The Hmisc package of the R program was utilized in order to do comparisons of the C-index between the nomogram and the staging systems developed by the American Joint Committee on Cancer. If the *p* values were lower than 0.05, then the null hypothesis, which states that there was no difference, was rejected.

### 2.11. Statistical Analysis

R (version 3.6.3) was used to carry out all statistical assays. The statistical analyses were performed on one-way analysis of variance (ANOVA) or two-tailed Student's *t*-test, and the results with a *p* value of less than 0.05 were determined as statistically significant.

## 3. Results

### 3.1. Pan-Cancer Analysis of GPD1L

**Figure 1:**
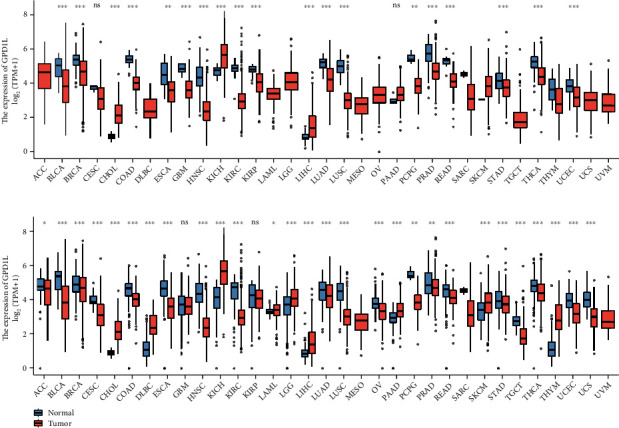
GPD1L levels that are either higher or lower in various malignancies when compared with normal tissues in (a) the TCGA datasets and (b) the TCGA and GTEx database. ^∗^*p* < 0.05, ^∗∗^*p* < 0.01, ^∗∗∗^*p* < 0.001; ns: no significance.

First, we carried out a pan-cancer study utilizing data from either TCGA or both TCGA and GTEx. Our research revealed that GPD1L demonstrated a dysregulated expression in a wide variety of malignancies, as illustrated in Figures 1(a) and 1(b). Furthermore, the expression pattern of GPD1L was shown to be variable in various types of cancers, which led researchers to hypothesize that GPD1L might act as tumor promoters or tumor suppressors.

### 3.2. The Expression of GPD1L in LUAD and Its Diagnostic Value

**Figure 2:**
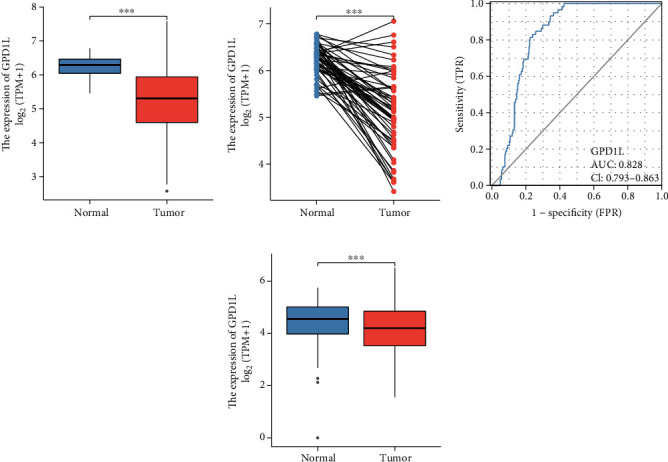
The levels of GPD1L expression in LUAD, as well as the diagnostic usefulness of this protein. (a, b) The levels of GPD1L expression found in LUAD specimens as compared to those found in nontumor tissues. (c) The ROC tests were utilized to evaluate the diagnostic potential of GPD1L. (d) The levels of GPD1L expression found in LUAD specimens compared to those found in non-tumor specimens, as determined by the TCGA or GTEx database. ^∗^*p* < 0.05, ^∗∗^*p* < 0.01, ^∗∗∗^*p* < 0.001; ns: no significance.

After that, we performed an analysis on the expression of GPD1L in LUAD and discovered that the expression of GPD1L was much lower in LUAD specimens when compared to specimens of nontumorous tissues (Figures 2(a) and 2(b)). Thereafter, the diagnostic utility of GPD1L was investigated further by us. The findings of ROC assays revealed that GPD1L was successful in differentiating LUAD specimens from normal specimens with an area under the ROC curves (AUC) of 0.828 (95% confidence interval: 0.793 to 0.863). These results are displayed in Figure 2(c). In addition, a discovery that is analogous to this one was discovered based on the TCGA and GTEx data (Figure 2(d)).

### 3.3. The Associations between GPD1L Expressions and Clinical Factors of LUAD Patients

**Figure 3:**
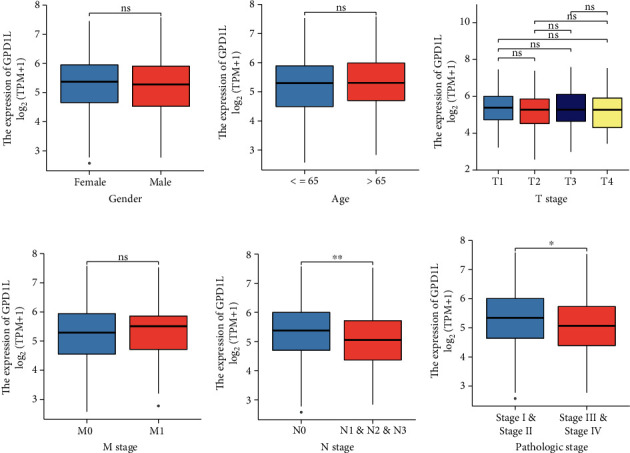
GPD1L expression and the clinical characteristics of LUAD patients have been found to have a correlation with one another. GPD1L expression was found to be associated with a number of clinicopathologic variables, such as (a) gender, (b) age, (c) T stage, (d) M stage, (e) N stage, and (f) pathologic stage. ^∗^*p* < 0.05, ^∗∗^*p* < 0.01, ^∗∗∗^*p* < 0.001; ns: no significance.

For the purpose of elucidating the function and importance of GPD1L expression, the TCGA data on all LUAD samples containing GPD1L expression data together with the characteristics of all patients were studied. As observed in Figures 3(a)–3(f) and Table 1, our investigation revealed that a low expression of GPD1L was inextricably linked to both the N stage and the clinical stage.

### 3.4. The Prognostic Values of GPD1L Expressions in LUAD

**Figure 4:**
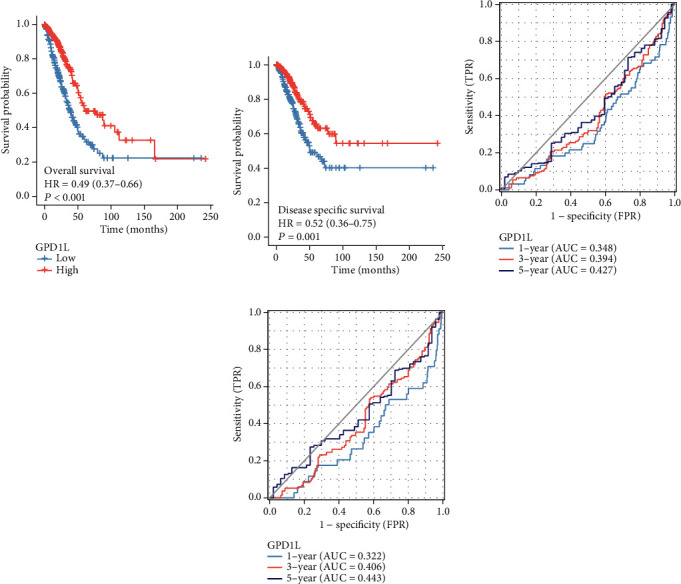
Analysis of the prognosis of GPD1L in patients with LUAD who were part of the TCGA cohort. Expression of GPD1L has been shown to have a correlation with both (a) overall survival and (b) survival specific to the disease. (c, d) The predictive performance of GPD1L expression in TCGA is evaluated using time-dependent receiver operating characteristic curves.

Through the use of survival analysis, we were able to further investigate whether or not GPD1L levels were connected with LUAD prognosis. According to the results of a Kaplan-Meier survival analysis, greater GPD1L expression predicted significantly improved OS (*p* < 0.001, Figure 4(a)) and DSS (*p* = 0.001, Figure 4(b)). GPD1L expression had a good predictive potential for the OS (AUC = 0.427, Figure 4(c)) and disease-specific survival (AUC = 0.443, Figure 4(d)) of LUAD patients, according to data from the TCGA. We performed univariate and multivariate analyses using Cox's proportional hazard model to further investigate the prognostic value of GPD1L expression in LUAD. Specifically, we demonstrated that the expression of GPD1L and the clinical stages were both independent prognostic indicators for overall survival (Table 2) and disease-specific survival (Table 3) in LUAD patients.

### 3.5. Construction and Validation of a Nomogram Based on the GPD1L Expression

**Figure 5:**
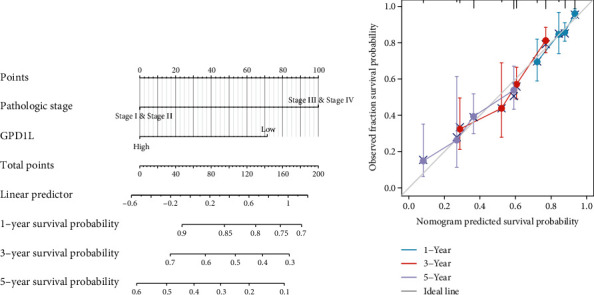
A quantitative method to predict the chance of 1-, 3-, and 5-year overall survival for patients diagnosed with LUAD. (a) A nomogram that estimates the likelihood of 1-, 3-, and 5-year overall survival for patients diagnosed with LUAD. (b) Calibration plots of the nomogram used to assess the overall likelihood of survival at 1, 3, and 5 years.

**Figure 6:**
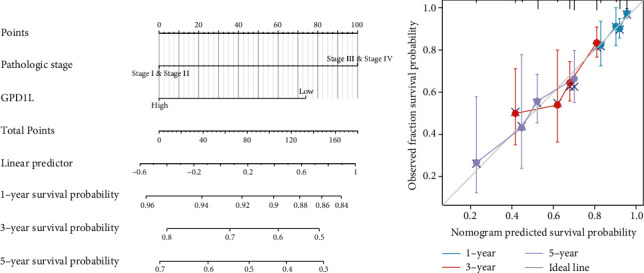
A quantitative method for estimating the likelihood that LUAD patients would develop the illness at 1, 3, and 5 years. (a) A nomogram that estimates the chance of disease-specific survival for LUAD patients at 1, 3, and 5 years. (b) Calibration plots of the nomogram used to assess the overall likelihood of survival at 1, 3, and 5 years.

In order to give a quantitative method for predicting the prognosis of LUAD patients, a nomogram was constructed using GPD1L and clinical stage as its two primary variables (Figure 5(a)). A point scale was utilized in the construction of the nomogram that was based on the multivariate Cox analysis. The variables were each given a certain number of points depending on the scale. When calculating the likelihood of surviving for LUAD patients at 1, 3, and 5 years, we drew a vertical line immediately downward from the total point axis to the outcome axis. Next, we also performed an analysis on the nomogram's ability to make accurate predictions, and the findings showed that the C-index of the model was 0.671 (CI: 0.650-0.691), which indicated that the nomogram's ability to make accurate predictions is only to a moderate degree (Figure 5(b)). In addition, we discovered a result that was comparable based on the DSS model (Figures 6(a) and 6(b)).

### 3.6. Functional Enrichment Analysis

**Figure 7:**
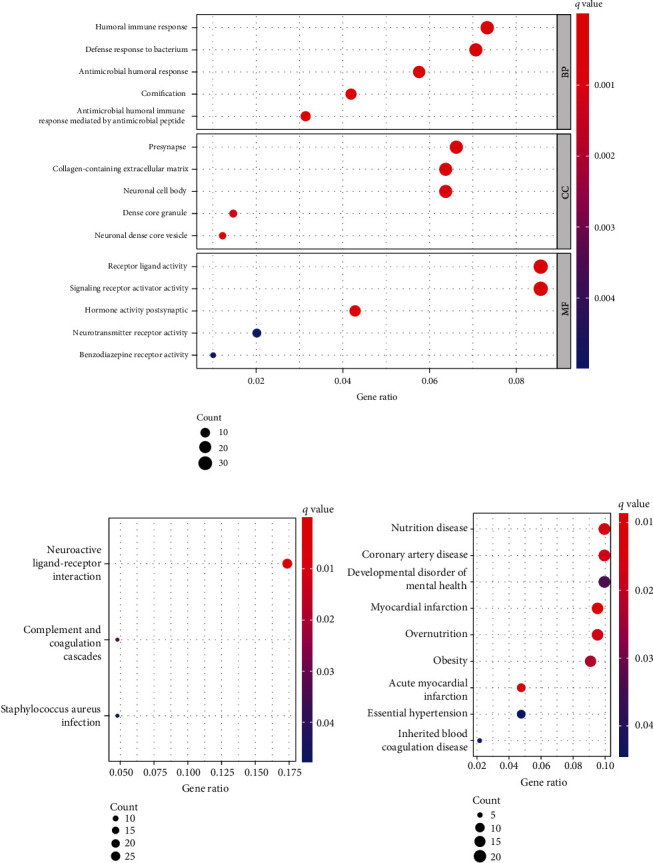
Functional enrichment analyses to identify potential biological processes. (a) The top 10 enriched BP, CC, and MF terms. (b) KEGG pathways. (c) Disease ontology enrichment analysis.

We first discovered a total of 454 DEGs. After that, we carried out a GO analysis with the 454 DEGs. As shown in Figure 7(a), we found that the 454 DEGs were mainly enriched in humoral immune response, defense response to the bacterium, antimicrobial humoral response, presynapse, neuronal cell body, dense core granule, receptor ligand activity, signaling receptor activator activity, and hormone activity. The results of KEGG revealed that the 454 DEGs were associated with neuroactive ligand-receptor interaction, complement and coagulation cascades, and *Staphylococcus aureus* infection (Figure 7(b)). In order to reveal more about the function of DEGs, an enrichment analysis of DO pathways was carried out. According to the findings, the majority of the disorders that were enriched by DEGs were related to nutrition disease, coronary artery disease, a developmental disorder of mental health, myocardial infarction, and overnutrition (Figure 7(c)).

### 3.7. The Expressions of GPD1L Were Associated with Immune Cell Infiltration

**Figure 8:**
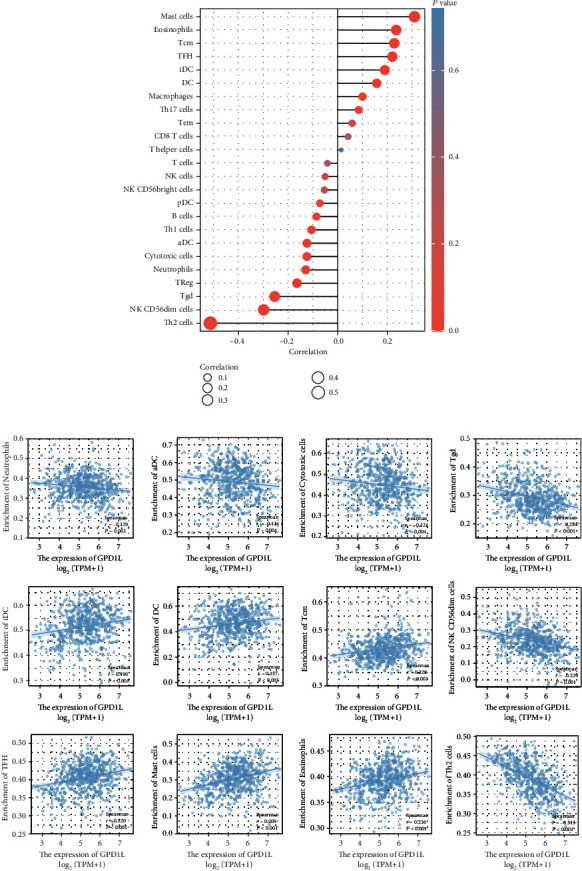
(a, b) Relationships between GPD1L and infiltrating immune cells in LUAD.

In order to evaluate the extent of immune cell infiltration that was present, the TCGA LUAD cohort's transcriptomes were analyzed using the ssGSEA methodology. This was done so that the researchers could determine the level of immune cell presence. In order to estimate the amount of immune cells that are present in the microenvironment of a tumor, the study included a total of twenty-four different words that were connected to the immune system. Our group observed that the expression of GPD1L was positively related to mast cells, eosinophils, Tcm, TFH, iDC, DC, and macrophages, while negatively associated with Th2 cells, NK CD56dim cells, Tgd, Treg, and neutrophils (Figures 8(a) and 8(b)).

### 3.8. Overexpression of GPD1L Suppressed the Proliferation of LUAD Cells

**Figure 9:**
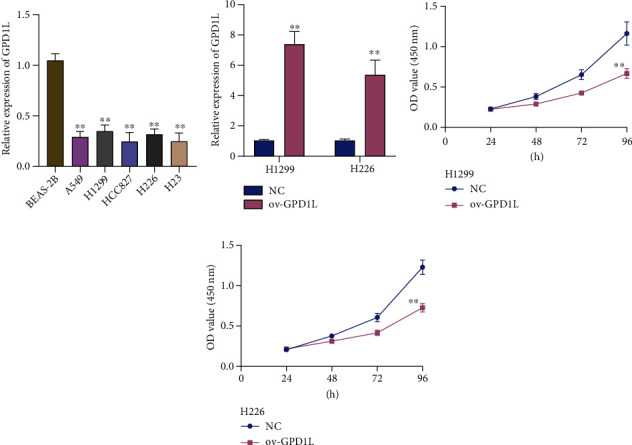
The overexpression of GPD1L in LUAD cells has been shown to limit cell growth. (a) The amount of GPD1L mRNA that is expressed relatively in LUAD cells (A549, H1299, HCC827, H226, and H23 cells) compared to BEAS-2B cells. (b) After treatment, there was an increase in the amount of GPD1L that was expressed in H1299 and H226. (c, d) The CCK-8 assay was utilized to determine whether or not the cells were viable. ^∗^*p* < 0.05, ^∗∗^*p* < 0.01, ^∗∗∗^*p* < 0.001; ns: no significance.

In order to provide more evidences for the presence of GPD1L in LUAD, we next carried out qRT-PCR examination and discovered that the expression of GPD1L was much lower in A549, H1299, HCC827, H226, and H23 cells when compared with BEAS-2B cells. This difference was rather noticeable (Figure 9(a)). In addition to this, it was demonstrated that treatment with GPD1L overexpressing plasmids (ov-GPD1L) resulted in a clear elevating expression of GPD1L (Figure 9(b)). Furthermore, we conducted CCK-8 tests, which enabled us to establish that the overexpression of GPD1L markedly inhibited the proliferation of H1299 and H226 cells (Figures 9(c) and 9(d)).

## 4. Discussion

In spite of the significant progress that has been made over the course of the past several years, LUAD continues to be regarded as a malignant tumor that has a dismal outlook when it is discovered at an advanced clinical stage [27, 28]. As a result, the investigation of the etiological factors and molecular mechanisms underlying LUAD is of the utmost significance for both treatment and prevention [29]. The quantity of data pertaining to genes has significantly expanded as a result of the ongoing development of gene chip and sequencing technology of the second generation [30, 31]. Therefore, one of the most pressing challenges facing researchers today is figuring out how to put these data to use to assist humans in better understanding the connection between genes and cancer.

The current study used data from the TCGA dataset to gather gene expression information. We discovered a new gene called GPD1L that was associated with cancer and found that its expression was aberrant in a wide variety of cancers. Previous researches had uncovered the roles that GPD1L played in a number of different cancers. For example, Tu et al. found that low levels of GPD1L expression in hepatocellular carcinoma were predictive of shorter overall survival times for patients with hepatocellular carcinoma [32]. Importantly, we discovered that the level of GPD1L expression was significantly lower in LUAD specimens compared to nontumor specimens, which suggested that it might play a role as a tumor suppressor gene in the evolution of LUAD. In addition, ROC assays demonstrated their diagnostic utility in screening LUAD specimens to differentiate them from nontumor specimens. The expression of GPD1L was shown to be an independent predictive factor for both overall survival and disease-free survival in the LUAD patients who were studied. Based on our findings, GPD1L may serve as a potential diagnostic and prognostic biomarker for patients suffering from LUAD.

After that, a total of 454 DEGs were discovered. Then, we carried out GO and KEGG assays and found that the 454 DEGs were primarily enriched in the following categories: antimicrobial humoral response; humoral immune response; defense response to bacterium; presynapse; neuronal cell body; dense core granule; receptor ligand activity; signaling receptor activator activity; hormone activity; and defense response to the bacterium. Based on our findings, GPD1L may be engaged in a number of different pathways that are associated with tumors.

TME can influence the development and progression of a tumor. In addition, it is made up of both cells that are part of the tumor and cells that are not part of the tumor, such as fibroblasts and immune cells [33, 34]. Immune cells that infiltrate tumors are strongly linked to angiogenesis and oncogenesis, as well as to the spread and proliferation of tumor cells [35, 36]. This connection may modulate the number of immune cells and how they differentiate. Recent researches had shed light on how inconsistencies between the advancement of a tumor and the immunological response of its host could contribute to the growth of the tumor [37]. TME was an essential component in both the beginning and the development of the tumorigenic process. Exploring the possible therapeutic targets that contribute to the remodeling of TME and supporting the transition of the TME from being tumor-friendly to being tumor-suppressed is of tremendous benefit [38, 39]. The significance of the immune microenvironment in the development of tumors was demonstrated by a significant number of research. Our findings from the study of the transcriptome based on the LUAD data in the TCGA database suggested that the immunological components present in the TME contributed to the prognosis of patients. Here, our group found that the expressions of GPD1L were positively associated with mast cells, eosinophils, Tcm, TFH, iDC, DC, and macrophages, while negatively associated with Th2 cells, NK CD56dim cells, Tgd, Treg, and neutrophils. Due to the fact that there was a correlation between the amounts of eosinophils, Th2 cells, and GPD1L expression in LUAD patients, it was shown that GPD1L might be responsible for the maintenance of an immune-active status in the TME.

Nevertheless, our investigation had a few drawbacks. First, we only used the data from the TCGA database for internal validation; in order to evaluate the applicability of the predictive signature, we require data from additional databases for external validation. Besides, experiments need to be conducted further in order to deeply uncover the mechanism of GPD1L in LUAD.

## 5. Conclusion

According to the results of our research, GPD1L expression is lower in patients with LUAD. Furthermore, the level of GPD1L expression is connected to the clinical case characteristics and prognosis of LUAD patients. The extent of immune cell infiltration, which may increase the antitumor impact, is directly tied to the level of expression of GPD1L. GPD1L is a biomarker that can be utilized in the diagnosis, treatment, and evaluation of the prognosis of LUAD.

## Figures and Tables

**Table 1:** The association between GPD1L expression and clinicopathological features.

Characteristic	Low expression of GPD1L	High expression of GPD1L	*p*
*n*	267	268	
Gender, *n* (%)			0.280
Female	136 (25.4%)	150 (28%)	
Male	131 (24.5%)	118 (22.1%)	
Age, *n* (%)			1.000
< =65	128 (24.8%)	127 (24.6%)	
>65	130 (25.2%)	131 (25.4%)	
Pathologic stage, *n* (%)			0.004
Stage I	128 (24.3%)	166 (31.5%)	
Stage II	72 (13.7%)	51 (9.7%)	
Stage III	52 (9.9%)	32 (6.1%)	
Stage IV	12 (2.3%)	14 (2.7%)	
T stage, *n* (%)			0.692
T1	81 (15.2%)	94 (17.7%)	
T2	150 (28.2%)	139 (26.1%)	
T3	25 (4.7%)	24 (4.5%)	
T4	10 (1.9%)	9 (1.7%)	
N stage, *n* (%)			0.002
N0	159 (30.6%)	189 (36.4%)	
N1	60 (11.6%)	35 (6.7%)	
N2	44 (8.5%)	30 (5.8%)	
N3	2 (0.4%)	0 (0%)	
M stage, *n* (%)			0.893
M0	186 (48.2%)	175 (45.3%)	
M1	12 (3.1%)	13 (3.4%)	
Age, median (IQR)	66 (58, 72)	66 (60, 73)	0.372

**Table 2:** Univariate and multivariate analysis of overall survival in LUAD patients.

Characteristics	Total (*n*)	Univariate analysis		Multivariate analysis	
		Hazard ratio (95% CI)	*p* value	Hazard ratio (95% CI)	*p* value
Gender	526				
Female	280	Reference			
Male	246	1.070 (0.803-1.426)	0.642		
Age	516				
< =65	255	Reference			
>65	261	1.223 (0.916-1.635)	0.172		
Pathologic stage	518				
Stage I and stage II	411	Reference			
Stage III and stage IV	107	2.664 (1.960-3.621)	<0.001	2.490 (1.829-3.391)	<0.001
GPD1L	526				
Low	263	Reference			
High	263	0.492 (0.365-0.662)	<0.001	0.521 (0.386-0.705)	<0.001

**Table 3:** Univariate and multivariate analysis of disease-specific survival in LUAD patients.

Characteristics	Total (*n*)	Univariate analysis		Multivariate analysis	
		Hazard ratio (95% CI)	*p* value	Hazard ratio (95% CI)	*p* value
Gender	491				
Female	262	Reference			
Male	229	0.989 (0.687-1.424)	0.954		
Age	481				
< =65	243	Reference			
>65	238	1.013 (0.701-1.464)	0.944		
Pathologic stage	483				
Stage I and stage II	389	Reference			
Stage III and stage IV	94	2.436 (1.645-3.605)	<0.001	2.269 (1.530-3.367)	<0.001
GPD1L	491				
Low	241	Reference			
High	250	0.517 (0.355-0.752)	<0.001	0.545 (0.373-0.798)	0.002

## Data Availability

All data are available from the corresponding authors under reasonable conditions.
